# Psychometric validation of the Chinese big five inventory-2 and its short form in adolescent football players

**DOI:** 10.3389/fpsyg.2026.1772587

**Published:** 2026-03-04

**Authors:** Ying Shuai, Shaoshen Wang, Garry Kuan, Yee Cheng Kueh

**Affiliations:** 1 School of Sports Management, Shandong Sport University, Jinan, Shandong Province, China; 2 Biostatistics & Research Methodology Unit, School of Medical Sciences, Universiti Sains Malaysia, Kubang Kerian, Kelantan, Malaysia; 3 Exercise and Sports Science Programme, School of Health Sciences, Universiti Sains Malaysia, Kubang Kerian, Kelantan, Malaysia

**Keywords:** adolescent athletes, cross-cultural validation, factor analysis, football, personality assessment, psychometric properties

## Abstract

**Background:**

This study examined the psychometric properties of the Chinese version of the Big Five Inventory-2 (BFI-2-C) and its short form (BFI-2-S-C) in adolescent football players. It addresses a critical gap in personality assessment within Chinese sport contexts.

**Methods:**

A cross-sectional validation study was conducted with 450 adolescent football players (52.2% male; mean age = 12.94 ± 0.78 years) from 24 schools across 12 urban centers in Shandong Province, China. Confirmatory factor analysis (CFA) using robust maximum-likelihood (MLR) estimation assessed structural validity. Reliability was evaluated through composite reliability coefficients and test–retest stability using Intraclass Correlation Coefficients. Construct validity examination included convergent validity (factor loadings, Average Variance Extracted, Composite Reliability) and discriminant validity (inter-factor correlations, Fornell-Larcker criterion).

**Results:**

Both instrument versions demonstrated excellent fit to the hypothesized five-factor structure. The original 60-item model exhibited robust indices (RMSEA = 0.029, 90% CI [0.026, 0.032]; CFI = 0.964; TLI = 0.962; SRMR = 0.036), while the 30-item abbreviated version yielded comparable or marginally superior parameters (RMSEA = 0.036, 90% CI [0.031, 0.042]; CFI = 0.971; TLI = 0.968; SRMR = 0.036). Reliability coefficients substantially exceeded conventional thresholds for both versions (CR = 0.923–0.950 and CR = 0.895–0.911, respectively). Test–retest stability ranged from good to excellent across all dimensions (ICC = 0.777–0.872 and ICC = 0.762–0.942, respectively). Convergent validity was established through strong factor loadings and acceptable AVE values, with the abbreviated version demonstrating enhanced convergent properties. Discriminant validity was confirmed through inter-factor correlations below critical thresholds and satisfaction of the Fornell-Larcker criterion.

**Conclusion:**

The BFI-2-C and BFI-2-S-C are valid and reliable instruments for assessing personality traits in Chinese adolescent football players. The short form provides a time-efficient alternative without compromising psychometric quality, making both tools suitable for use in sport psychology research and applied youth athlete development.

## Introduction

Personality, defined as consistent patterns of thought, emotion, and behavior that demonstrate relative stability across diverse contexts and temporal landscapes, offers a fundamental lens through which to understand human uniqueness (Burger and Reevy, 2022). In applied settings such as youth sport development, understanding personality traits is particularly valuable as these enduring dispositions influence key outcomes including performance consistency, adherence to training, stress management, and psychosocial adjustment (Cervone and Pervin, 2022).

The measurement of personality in specialized populations, such as adolescent athletes, presents unique methodological considerations. Adolescence represents a critical developmental period characterized by significant physical, psychological, and social transitions (Proctor and Capaldi, 2001). When combined with the distinctive demands of competitive sport environments—including intensive training regimens, performance pressures, and team dynamics—the assessment of personality traits requires psychometrically validated instruments that demonstrate reliability and validity within these specific contexts (Allen et al., 2013; Côté et al., 2007). Establishing the psychometric adequacy of personality assessment tools in youth athletic populations is therefore essential for both research validity and applied intervention effectiveness.

The scholarly exploration of personality is characterized by a rich tapestry of theoretical perspectives. No single framework—be it Freudian psychodynamics, phenomenological approaches, trait theories, behaviorism, or social-cognitive paradigms—can exhaustively capture the multifaceted nature of human personality. Instead, these theories are more aptly conceived as complementary conceptual instruments, each offering unique methodological approaches and explanatory frameworks (Proctor and Capaldi, 2001). This theoretical pluralism fosters continuous refinement and empirical advancement, thereby expanding our collective understanding of personality (Cervone and Pervin, 2022).

Within contemporary personality research, the Five-Factor Model (FFM), or Big Five model, has emerged as a dominant and extensively validated framework since gaining prominence in the 1980s (John and Srivastava, 1999; McCrae and Costa, 2008). This model parsimoniously organizes personality structure into five broad, relatively independent dimensions: Extraversion (sociable, assertive, energetic vs. solitary, reserved), Agreeableness (compassionate, respectful vs. antagonistic, critical), Conscientiousness (organized, productive vs. careless, inefficient), Negative Emotionality (or Neuroticism; anxious, emotionally volatile vs. calm, composed), and Open-Mindedness (or Openness to Experience; intellectually curious, creative vs. conventional, routine-oriented; Roccas et al., 2002; Soto and John, 2017a). Factor-analytic studies of trait descriptors in natural language and personality questionnaire items have consistently revealed these five factors, reflecting their robustness and cross-cultural relevance (Goldberg, 1993). These dimensions, often recalled by the acronyms “OCEAN” or “CANOE,” are conceptualized as spectra rather than categorical typologies, offering a nuanced depiction of individual differences (De Bolle et al., 2012; Matthews et al., 2003).

To operationalize these Big Five traits, various assessment instruments have been developed. Among the most contemporary and methodologically rigorous is the Big Five Inventory-2 (BFI-2), developed by Soto and John (2017a). The BFI-2 is a 60-item self-report measure that significantly revises and extends the original BFI (John et al., 1991). A key methodological strength of the BFI-2 is its hierarchical structure, assessing not only the five broad domains but also 15 more specific facet traits (three facets nested within each domain). This hierarchical approach enhances both conceptual breadth at the domain level and specificity at the facet level, leading to greater bandwidth, fidelity, and predictive power compared to its predecessor (Soto and John, 2017a). Furthermore, the BFI-2 implements equal numbers of true-keyed and false-keyed items for each scale, effectively controlling for acquiescent responding bias—a methodological consideration vital for cross-cultural research (Soto and John, 2017a).

For research contexts with time constraints, Soto and John developed validated short forms of the BFI-2 (Soto and John, 2017b). The BFI-2 measurement system now includes multiple abbreviated versions to accommodate varying research needs: the BFI-2 Short Form (BFI-2-S) comprises 30 items measuring both domains and facets, the BFI-2 Extra-Short Form (BFI-2-XS) offers a 15-item domain-level assessment, and the BFI-2 Ultra-Short Form provides a 10-item assessment for extremely time-constrained contexts (Soto and John, 2017a; Soto and John, 2017b). Although the full BFI-2 is generally recommended for superior psychometric properties, these abbreviated versions provide valuable alternatives for large-scale research protocols where administration time is limited, with each version balancing psychometric rigor against practical constraints (Soto and John, 2017b).

The BFI-2 measurement system has undergone extensive cross-cultural validation across diverse linguistic and cultural contexts. European validations include Denmark (Vedel et al., 2020), Germany (Danner et al., 2016) and France (Lignier et al., 2023). In Asian contexts, validations have been conducted in Japan (Yoshino et al., 2022), Indonesia (Yoshino et al., 2022), and China (Zhang et al., 2022). Specifically, Zhang, Li (Zhang et al., 2022) conducted a comprehensive psychometric evaluation of the full 60-item Chinese BFI-2 (BFI-2-C) across four diverse samples, including college students, adult employees, adults in substance use treatment, and general adolescents. Their findings largely supported the reliability, structural validity, and criterion-related validity of the Chinese BFI-2 at both the domain level (five broad factors) and the facet level (15 specific facets), although certain facets and negatively worded items performed better among participants with higher education levels (Zhang et al., 2022). Notably, while Zhang et al.’s validation included a general adolescent sample, no prior validation has examined the psychometric properties of either the full BFI-2-C or its 30-item short form (BFI-2-S-C) specifically within specialized youth athletic populations. These international validation studies underscore the cross-cultural applicability of both the Big Five model and its measurement instruments.

The assessment of personality traits is particularly salient in applied domains such as sports psychology. Personality characteristics influence numerous sport-related outcomes, including athletic performance, adherence to training regimens, stress-coping strategies, team cohesion, and leadership (Allen et al., 2013; Laborde et al., 2019). Specific traits such as Conscientiousness, Extraversion, or low Negative Emotionality have been linked to better adaptation, resilience, and success in competitive sports environments (Allen et al., 2011; Shuai et al., 2023). However, the applied utility of personality assessment is fundamentally contingent upon the psychometric adequacy of the measurement instruments employed. The conceptual importance of personality in sport does not automatically guarantee that instruments validated in general populations will demonstrate equivalent measurement properties in specialized athletic contexts (Laborde et al., 2016). From a psychometric perspective, establishing measurement equivalence—that is, demonstrating that an instrument functions similarly across different populations—is a prerequisite for valid score interpretation and meaningful cross-group comparisons (Cheung and Rensvold, 2002). Without empirical validation demonstrating factorial invariance, adequate reliability, and construct validity within a specific population, the application of personality assessments risks measurement error, construct irrelevance, and potentially misleading conclusions regarding individual differences (Skorupiński, 2015).

Despite the growing body of research on the BFI-2, including its validation in general Chinese adolescent populations (Zhang et al., 2022), empirical evidence suggests that psychometric properties of personality instruments may not automatically generalize across specialized subpopulations, particularly those experiencing distinctive developmental contexts and performance demands. Specifically, the psychometric properties of the BFI-2, and by extension its widely used 30-item short form (BFI-2-S), have not been systematically investigated among Chinese adolescent football players. Several lines of empirical evidence support the necessity of population-specific validation in youth sport contexts. First, prior research has documented measurement non-invariance in personality assessments across athlete and non-athlete populations, suggesting that the factorial structure and item functioning may differ systematically (Laborde et al., 2016; Laborde et al., 2017). Second, studies examining trait emotional intelligence and other psychological constructs have found that specialized athletic populations exhibit different response patterns and psychometric properties compared to general populations, even when controlling for age and cultural background (Laborde et al., 2016). Third, research on Chinese youth sport development has highlighted unique sociocultural factors—including collectivist team dynamics, early specialization pressures, and sport-specific achievement orientations—that may influence both personality expression and self-report patterns (Bergeron et al., 2015; Cheng, 2021).

Adolescent football players in China operate within a unique developmental ecosystem characterized by intensive training regimens (often 15–20 h weekly), high-stakes competitive pressures from early ages, hierarchical team structures, and institutional expectations specific to the Chinese youth football development system (Cheng, 2021). These contextual factors may systematically influence how personality traits are manifested, perceived, and reported through self-assessment, potentially affecting the validity and reliability of personality assessment instruments originally validated in general populations (Laborde et al., 2016; Laborde et al., 2017). Without empirical verification, assuming measurement equivalence across such distinct contexts risks compromising both research validity and the appropriateness of applied interventions.

Establishing the psychometric integrity of the BFI-2 and BFI-2-S within this cohort is methodologically imperative for several reasons: (1) it would provide researchers and practitioners with a culturally and contextually validated assessment tool for understanding personality in young Chinese football talents; (2) it could inform evidence-based practices in talent identification, player development programs, and psychological support interventions; and (3) it would contribute to a more nuanced understanding of personality-performance relationships in a globally popular sport within a major athletic nation.

Therefore, the primary aim of the present study is to rigorously investigate the psychometric properties of the Big Five Inventory-2 (BFI-2) in a sample of Chinese adolescent football players. This investigation encompasses comprehensive analyses of reliability, factor structure, and construct validity. Furthermore, given the practical advantages of briefer assessment instruments in applied sport settings, this study also examines the psychometric characteristics and applicability of the Big Five Inventory-2 Short Form (BFI-2-S). Such methodologically rigorous validation addresses a significant lacuna in the literature and provides a foundational framework for future personality research and application in Chinese youth football development.

## Materials and methods

### Participants

This investigation employed a cross-sectional validation design to evaluate the psychometric properties of the Chinese version of the Big Five Inventory-2 (BFI-2 -C) and Big Five Inventory-2-Short (BFI-2-S-C). The reference population encompassed adolescent football players aged 12–15 years from Shandong Province, China, who were actively engaged in football training and competitive activities either through school programs or club affiliations. Participant recruitment occurred across middle schools in 12 urban centers within Shandong Province, with specific focus on institutions recognized for their established football programs and institutional willingness to participate in research initiatives. Consequently, the source population constituted a specific subset of the broader reference population. The sampling framework utilized official football player registries provided by participating educational institutions, which were collaboratively developed by administrative personnel and football coaching staff to ensure all registered players fulfilled inclusion parameters.

Eligibility criteria for participation included: (1) Chinese nationality; (2) aged 12–15 years; (3) enrolled in participating junior high schools in Shandong Province; (4) active participation in school football teams with at least 1 year of training experience; (5) sufficient Chinese language comprehension and literacy; (6) voluntary participation with both individual assent and parental consent obtained.

Sample size requirements for confirmatory factor analysis were calculated utilizing WN Arifin’s web-based computational tool, incorporating parameters established in contemporary structural equation modeling literature (Arifin, 2026). The calculation integrated the following specifications: anticipated Comparative Fit Index (CFI) of 0.95, five-factor structure with 12 items per factor, expected factor loadings of 0.6, inter-factor correlations of 0.25, significance threshold (*α*) of 0.05 (two-tailed), and desired statistical power (1–*β*) of 95%. After accounting for potential participant attrition (20%), the minimum required sample was determined to be 433 participants. The actual sample size obtained (*N* = 450) substantially exceeded this requirement, ensuring robust parameter estimation and factor solution stability.

### Measures

#### The big five inventory–2

The BFI-2 assesses individual differences across multiple personality dimensions. The instrument administered in this investigation comprised 60 items measuring five personality domains: extraversion, agreeableness, conscientiousness, negative emotionality, and open-mindedness. Each item utilizes a 5-point Likert scale ranging from 1 (strongly disagree) to 5 (strongly agree). Previous validation studies have demonstrated sound psychometric properties for the BFI-2, with internal consistency coefficients (Cronbach’s alpha) ranging from 0.53 to 0.80 across subscales. Construct validity assessment through confirmatory factor analysis revealed adequate model fit indices (CFI = 0.97–0.99, TLI = 0.91–0.98, RMSEA = 0.03–0.06) in the original validation research (Cieslak, 2004).

### Ethics and procedures

The study received ethical approval from the Universiti Sains Malaysia Human Research Ethics Committee (USM/JEPeM/22050288). The adaptation of the BFI-2 into Chinese (BFI-2-C) followed a rigorous six-stage translation and cross-cultural adaptation process adhering to established international guidelines (Beaton et al., 2000; Sousa and Rojjanasrirat, 2011).

First, two independent bilingual translators, both proficient in English and Chinese and knowledgeable about Chinese sport contexts, performed forward translations from English to Chinese. One translator possessed expertise in psychological assessment, while the other was a sport psychologist specializing in youth athletics. Second, the research team synthesized these two forward translations into a single preliminary Chinese version, resolving any discrepancies through discussion until consensus was achieved. Third, two different bilingual translators, blind to the original English version, independently conducted back-translations from Chinese to English. Both back-translators were native Chinese speakers with experience in psychological research. Fourth, an expert committee comprising all four translators, the research team, and two additional experts in sport psychology and psychometrics reviewed all translations. The committee compared the back-translated versions with the original English instrument to ensure semantic, idiomatic, experiential, and conceptual equivalence. Any discrepancies were discussed until consensus was reached on the final Chinese wording. Fifth, cognitive debriefing was conducted with 20 adolescent football players (10 boys, 10 girls) not included in the main study sample. Participants completed the translated BFI-2-C and provided feedback regarding item clarity, relevance, and cultural appropriateness. Based on their feedback, minor linguistic adjustments were made to enhance comprehension while maintaining conceptual fidelity to the original instrument. Sixth, following incorporation of debriefing feedback, the research team conducted a final comprehensive review, and a professional proofreader examined the instrument for grammatical and typographical accuracy. This systematic adaptation process ensured that the Chinese BFI-2-C maintained semantic accuracy, cultural relevance, and conceptual equivalence with the original English version (Beaton et al., 2000).

Data were collected from October to December 2023 via face-to-face administration at participating schools. Information sessions were held at each school to explain the study’s objectives, procedures, confidentiality, and voluntary nature to potential participants and their parents/guardians. Written informed consent was obtained from the parents or legal guardians of all participants, and written assent was obtained from the adolescent participants themselves.

Questionnaire administration took place in controlled classroom environments under the supervision of trained research personnel. Standardized instructions were provided, and participants were encouraged to seek clarification if needed. Research assistants maintained availability for question resolution while ensuring a non-intrusive presence to facilitate independent response patterns. Participants received assurances regarding response anonymity and confidentiality. Completed instruments were immediately collected and reviewed for completeness. For test–retest reliability assessment, a subsample of 50 participants from Shandong Luneng Taishan Football School completed a follow-up assessment after a two-week interval. This sample size exceeds the minimum recommendation of 30–50 participants for test–retest reliability studies (Koo and Li, 2016) and provides adequate statistical power for estimating intraclass correlation coefficients with acceptable precision (Walter et al., 1998). The two-week interval was selected to minimize both memory effects and genuine trait changes (Streiner et al., 2024). All retest assessments were conducted under controlled environmental conditions identical to the initial administration to minimize confounding variables.

### Statistical analysis

Preliminary data screening examined distributional properties through SPSS 28.0 (IBM Corp, Armonk, NY, United States). Univariate normality was assessed using Kolmogorov–Smirnov and Shapiro–Wilk procedures, while multivariate normality evaluation employed Mardia’s coefficients for skewness and kurtosis (Ghasemi and Zahediasl, 2012). Visual examination of histograms and chi-square versus Mahalanobis distance plots supplemented these analyses following established methodological recommendations (Tabachnick et al., 2019).

Confirmatory Factor Analysis was implemented through Mplus 8.7 utilizing Maximum Likelihood Robust (MLR) estimation. MLR was selected based on preliminary assumption checking which indicated departures from normality. MLR is appropriate for Likert-scale data with five or more response categories and is robust to non-normality (Rhemtulla et al., 2012; Li, 2016). Model adequacy was evaluated through multiple fit indices with established thresholds: Comparative Fit Index (Hu and Bentler, 1999): Comparative Fit Index (CFI > 0.95), Tucker-Lewis Index (TLI > 0.95), Root Mean Square Error of Approximation (RMSEA < 0.06, with 90% confidence intervals), and Standardized Root Mean Square Residual (SRMR < 0.08). Factor loadings exceeding 0.40 were considered acceptable based on established guidelines (Hair et al., 2012).

Prior to CFA, distributional properties were examined to determine the appropriate estimation method. Kolmogorov–Smirnov and Shapiro–Wilk tests indicated significant departures from univariate normality for all 60 items (all *p* < 0.001). Visual inspection of histograms confirmed non-normal distributions for item scores.

Multivariate normality assessment using Mplus 8.7 revealed significant departures from both multivariate skewness and kurtosis. The two-sided multivariate skew test yielded: sample value = 544.567, mean = 501.239, standard deviation = 4.052, *p* < 0.001. The two-sided multivariate kurtosis test yielded: sample value = 3766.433, mean = 3703.656, standard deviation = 6.428, *p* < 0.001. Visual examination of chi-square versus Mahalanobis distance plots showed deviation from linearity, further confirming violation of multivariate normality assumptions.

All variance–covariance matrices were positive definite, indicating absence of multicollinearity issues. Given the departures from normality but the use of 5-point Likert scales, MLR estimation was selected for its robustness to non-normality while accommodating the continuous treatment of ordinal data with five or more categories (Rhemtulla et al., 2012; Li, 2016).

Construct validity assessment incorporated both convergent and discriminant validity analyses. Convergent validity was established through examination of factor loadings (> 0.50), Average Variance Extracted (AVE > 0.50), and Composite Reliability (CR > 0.70) following recommended methodological protocols (Hair et al., 2012). Discriminant validity evaluation employed the Fornell-Larcker criterion, requiring the square root of AVE for each construct to exceed its correlations with other measured constructs (Fornell and Larcker, 1981).

Reliability assessment encompassed both internal consistency and temporal stability measures. Internal consistency was evaluated through Composite Reliability coefficients, with values exceeding 0.70 indicating satisfactory reliability (Taber, 2018). Test–retest reliability was assessed using two-way mixed effects Intraclass Correlation Coefficients, with resultant values interpreted according to established guidelines: insufficient (< 0.50), moderate (0.50–0.75), good (0.75–0.90), and excellent (> 0.90; Koo and Li, 2016).

## Results

### Descriptive statistics

Table 1 presents the demographic characteristics of the CFA participants (*N* = 450). The mean age of the participants was 12.94 years (SD = 0.78), indicating that the sample primarily consisted of young adolescents. The gender distribution was relatively balanced, with 52.2% male (*n* = 235) and 47.8% female (*n* = 215) participants. Participants were recruited from 12 cities across Shandong Province, with the largest proportions coming from Weifang (15.3%), Linyi (13.1%), and Zibo (10%). The sample included students from 24 different schools, with the largest group (11.1%) from Shandong Luneng Taishan Football School. In terms of academic grade, the majority of participants were in Grade 2 of junior high school (52.7%), followed by Grade 1 (34.4%) and Grade 3 (12.9%). Regarding their positions on the football field, the sample included a mix of defenders (39.3%), midfielders (26.2%), forwards (25.1%), and goalkeepers (9.3%).

**Table 1:** Demographic information and frequency of participants.

Category	Name	Frequency	Percent	Mean (SD)
Age				12.94 (0.78)
City	Binzhou	19	4.2	
	Dongying	33	7.3	
	Heze	18	4.0	
	Jinan	37	8.2	
	Jining	28	6.2	
	Liaocheng	35	7.8	
	Linyi	59	13.1	
	Qingdao	41	9.1	
	Tai’an	38	8.4	
	Weifang	69	15.3	
	Zaozhuang	28	6.2	
	Zibo	45	10.0	
School	Binzhou Development Zone No.2 Middle School	19	4.2	
	Chengyang Experiment	19	4.2	
	Dianliu No.1 Middle School	19	4.2	
	Dongying Experiment	33	7.3	
	Heze Caozhou Military School	18	4.0	
	Jining University Affiliated Middle School	15	3.3	
	Liaocheng Shaolin	17	3.8	
	Linyi Phoenix Experiment	13	2.9	
	Linyi No.16 Middle School	14	3.1	
	Linzi Experiment	26	5.8	
	Shandong Luneng Taishan Football School	50	11.1	
	Qingdao Chengyang	22	4.9	
	Qingzhou Banner City	19	4.2	
	Shifeng Middle School	18	4.0	
	Tai’an Kaiyuan	18	4.0	
	Tai’an No.1 Middle School	20	4.4	
	Tancheng Nurturing Talents	18	4.0	
	Tangye Middle School	18	4.0	
	Wenshang Experiment	13	2.9	
	Yinan Huate Wolong	14	3.1	
	Zaozhuang No.15 Middle School	14	3.1	
	Zaozhuang Experiment	14	3.1	
	Zhangdian No.8 Middle School	13	2.9	
	Zibo No.5 Middle School	6	1.3	
Gender	Male	235	52.2	
	Female	215	47.8	
Grade	Grade 1 of junior high school	155	34.4	
	Grade 2 of junior high school	237	52.7	
	Grade 3 of junior high school	58	12.9	
Position	Forward	113	25.1	
	Midfielder	118	26.2	
	Defender	177	39.3	
	Goalkeeper	42	9.3	

SD, Standard Deviation.

Table 2 shows the score distribution for the 60 items of the BFI2-C (Chinese) scale. The scores range from 1 (strongly disagree) to 5 (strongly agree). Most items have mean scores between 3 and 4, indicating generally neutral to slightly positive responses. The highest mean score was 4.4 (SD = 0.88) for item BFI17, with 60.2% of respondents choosing “strongly agree.” The lowest mean score was 2.69 (SD = 1.13) for item BFI20, with 45.2% of respondents choosing either “disagree” or “strongly disagree.” The distribution of scores for each item in the BFI2-C scale were generally normally distributed, with a slight skew toward higher scores for some items.

**Table 2:** Distribution of the items’ score for Chinese version of BFI-2-C scale.

Items	1 n(%)	2 n(%)	3 n(%)	4 n(%)	5 n(%)	Mean (SD)
BFI1	6 (1.3)	40 (8.9)	128 (28.4)	142 (31.6)	134 (29.8)	3.80 (1.01)
BFI2	2 (0.4)	18 (4.0)	127 (28.2)	173 (38.4)	130 (28.9)	3.91 (0.87)
BFI3	19 (4.2)	66 (14.7)	135 (30.0)	161 (35.8)	69 (15.3)	3.43 (1.05)
BFI4	24 (5.3)	62 (13.8)	169 (37.6)	127 (28.2)	68 (15.1)	3.34 (1.06)
BFI5	38 (8.4)	50 (11.1)	126 (28.0)	148 (32.9)	88 (19.6)	3.44 (1.17)
BFI6	17 (3.8)	51 (11.3)	134 (29.8)	133 (29.6)	115 (25.6)	3.62 (1.10)
BFI7	5 (1.1)	10 (2.2)	77 (17.1)	167 (37.1)	191 (42.4)	4.18 (0.87)
BFI8	25 (5.6)	67 (14.9)	144 (32.0)	131 (29.1)	83 (18.4)	3.40 (1.11)
BFI9	10 (2.2)	41 (9.1)	124 (27.6)	163 (36.2)	112 (24.9)	3.72 (1.01)
BFI10	13 (2.9)	42 (9.3)	119 (26.4)	142 (31.6)	134 (29.8)	3.76 (1.07)
BFI11	25 (5.6)	45 (10.0)	137 (30.4)	164 (36.4)	79 (17.6)	3.50 (1.07)
BFI12	9 (2.0)	18 (4.0)	67 (14.9)	172 (38.2)	184 (40.9)	4.12 (0.94)
BFI13	16 (3.6)	21 (4.7)	156 (34.7)	155 (34.4)	102 (22.7)	3.68 (0.99)
BFI14	39 (8.7)	74 (16.4)	120 (26.7)	139 (30.9)	78 (17.3)	3.32 (1.19)
BFI15	5 (1.1)	46 (10.2)	184 (40.9)	143 (31.8)	72 (16.0)	3.51 (0.92)
BFI16	44 (9.8)	88 (19.6)	147 (32.7)	107 (23.8)	64 (14.2)	3.13 (1.17)
BFI17	6 (1.3)	13 (2.9)	46 (10.2)	114 (25.3)	271 (60.2)	4.40 (0.88)
BFI18	15 (3.3)	30 (6.7)	185 (41.1)	150 (33.3)	70 (15.6)	3.51 (0.95)
BFI19	58 (12.9)	118 (26.2)	158 (35.1)	87 (19.3)	29 (6.4)	2.80 (1.09)
BFI20	70 (15.6)	133 (29.6)	147 (32.7)	67 (14.9)	33 (7.3)	2.69 (1.13)
BFI21	5 (1.1)	30 (6.7)	137 (30.4)	180 (40.0)	98 (21.8)	3.75 (0.91)
BFI22	8 (1.8)	28 (6.2)	133 (29.6)	190 (42.2)	91 (20.2)	3.73 (0.91)
BFI23	8 (1.8)	27 (6.0)	101 (22.4)	174 (38.7)	140 (31.1)	3.91 (0.96)
BFI24	29 (6.4)	90 (20.0)	192 (42.7)	92 (20.4)	47 (10.4)	3.08 (1.04)
BFI25	21 (4.7)	51 (11.3)	173 (38.4)	132 (29.3)	73 (16.2)	3.41 (1.04)
BFI26	13 (2.9)	33 (7.3)	99 (22.0)	152 (33.8)	153 (34)	3.89 (1.05)
BFI27	15 (3.3)	25 (5.6)	182 (40.4)	148 (32.9)	80 (17.8)	3.56 (0.96)
BFI28	15 (3.3)	49 (10.9)	148 (32.9)	151 (33.6)	87 (19.3)	3.55 (1.03)
BFI29	37 (8.2)	89 (19.8)	121 (26.9)	111 (24.7)	92 (20.4)	3.29 (1.23)
BFI30	6 (1.3)	22 (4.9)	108 (24.0)	187 (41.6)	127 (28.2)	3.90 (0.91)
BFI31	66 (14.7)	74 (16.4)	124 (27.6)	118 (26.2)	68 (15.1)	3.11 (1.27)
BFI32	3 (0.7)	17 (3.8)	160 (35.6)	151 (33.6)	119 (26.4)	3.81 (0.89)
BFI33	7 (1.6)	33 (7.3)	145 (32.2)	153 (34.0)	112 (24.9)	3.73 (0.97)
BFI34	71 (15.8)	108 (24.0)	125 (27.8)	110 (24.4)	36 (8.0)	2.85 (1.19)
BFI35	35 (7.8)	110 (24.4)	166 (36.9)	86 (19.1)	53 (11.8)	3.03 (1.10)
BFI36	27 (6.0)	69 (15.3)	200 (44.4)	114 (25.3)	40 (8.9)	3.16 (0.99)
BFI37	28 (6.2)	98 (21.8)	132 (29.3)	122 (27.1)	70 (15.6)	3.24 (1.14)
BFI38	14 (3.1)	51 (11.3)	181 (40.2)	126 (28.0)	78 (17.3)	3.45 (1.00)
BFI39	39 (8.7)	83 (18.4)	125 (27.8)	127 (28.2)	76 (16.9)	3.26 (1.19)
BFI40	13 (2.9)	31 (6.9)	220 (48.9)	127 (28.2)	59 (13.1)	3.42 (0.90)
BFI41	2 (0.4)	24 (5.3)	134 (29.8)	174 (38.7)	116 (25.8)	3.84 (0.89)
BFI42	11 (2.4)	42 (9.3)	123 (27.3)	177 (39.3)	97 (21.6)	3.68 (0.99)
BFI43	11 (2.4)	32 (7.1)	202 (44.9)	136 (30.2)	69 (15.3)	3.49 (0.92)
BFI44	19 (4.2)	39 (8.7)	132 (29.3)	162 (36.0)	98 (21.8)	3.62 (1.05)
BFI45	16 (3.6)	40 (8.9)	109 (24.2)	177 (39.3)	108 (24.0)	3.71 (1.04)
BFI46	23 (5.1)	55 (12.2)	159 (35.3)	109 (24.2)	104 (23.1)	3.48 (1.13)
BFI47	15 (3.3)	41 (9.1)	122 (27.1)	143 (31.8)	129 (28.7)	3.73 (1.07)
BFI48	12 (2.7)	11 (2.4)	62 (13.8)	148 (32.9)	217 (48.2)	4.22 (0.96)
BFI49	47 (10.4)	109 (24.2)	155 (34.4)	93 (20.7)	46 (10.2)	2.96 (1.13)
BFI50	52 (11.6)	62 (13.8)	157 (34.9)	104 (23.1)	75 (16.7)	3.20 (1.21)
BFI51	23 (5.1)	67 (14.9)	196 (43.6)	115 (25.6)	49 (10.9)	3.22 (1.00)
BFI52	10 (2.2)	17 (3.8)	147 (32.7)	166 (36.9)	110 (24.4)	3.78 (0.93)
BFI53	8 (1.8)	30 (6.7)	138 (30.7)	167 (37.1)	107 (23.8)	3.74 (0.95)
BFI54	29 (6.4)	65 (14.4)	162 (36)	125 (27.8)	69 (15.3)	3.31 (1.10)
BFI55	30 (6.7)	59 (13.1)	161 (35.8)	133 (29.6)	67 (14.9)	3.33 (1.09)
BFI56	4 (0.9)	20 (4.4)	112 (24.9)	161 (35.8)	153 (34.0)	3.98 (0.92)
BFI57	8 (1.8)	29 (6.4)	158 (35.1)	149 (33.1)	106 (23.6)	3.70 (0.96)
BFI58	22 (4.9)	70 (15.6)	146 (32.4)	139 (30.9)	73 (16.2)	3.38 (1.08)
BFI59	34 (7.6)	56 (12.4)	132 (29.3)	126 (28.0)	102 (22.7)	3.46 (1.19)
BFI60	7 (1.6)	40 (8.9)	174 (38.7)	162 (36.0)	67 (14.9)	3.54 (0.91)

1 = strongly disagree, 2 = disagree, 3 = neutral, 4 = agree, 5 = strongly agree. SD, standard deviation.

### Confirmatory factor analysis

**Figure 1:**
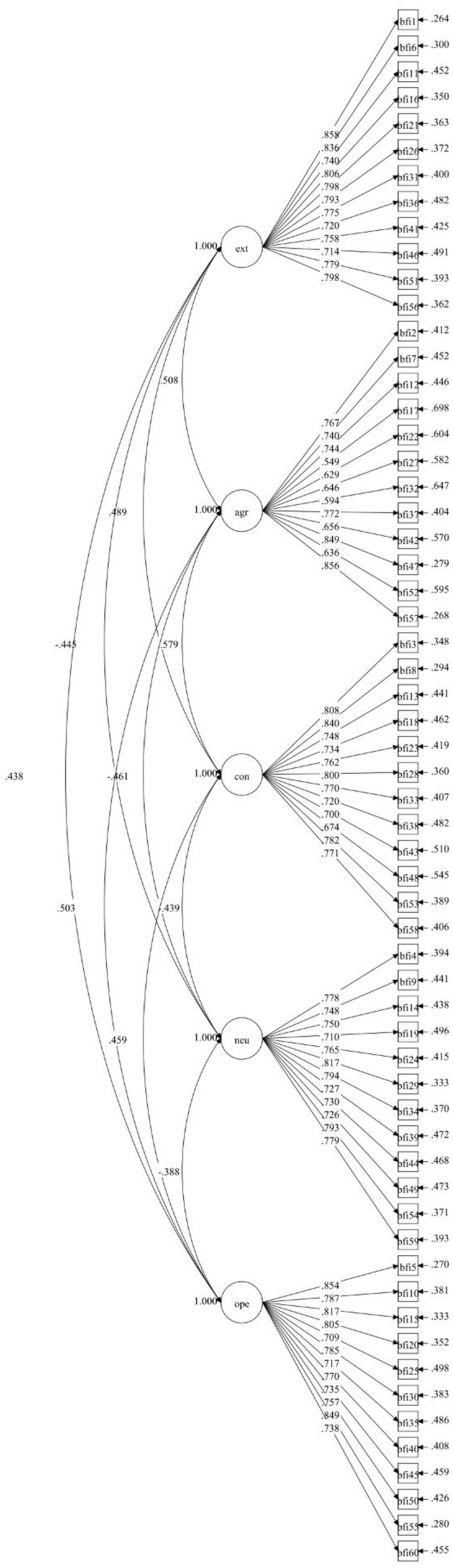
CFA diagram of the BFI-2-C original model. EXT, extraversion; AGR, agreeableness; CON, conscientiousness; NEU, negative emotionality; OPE, open-mindedness.

**Figure 2:**
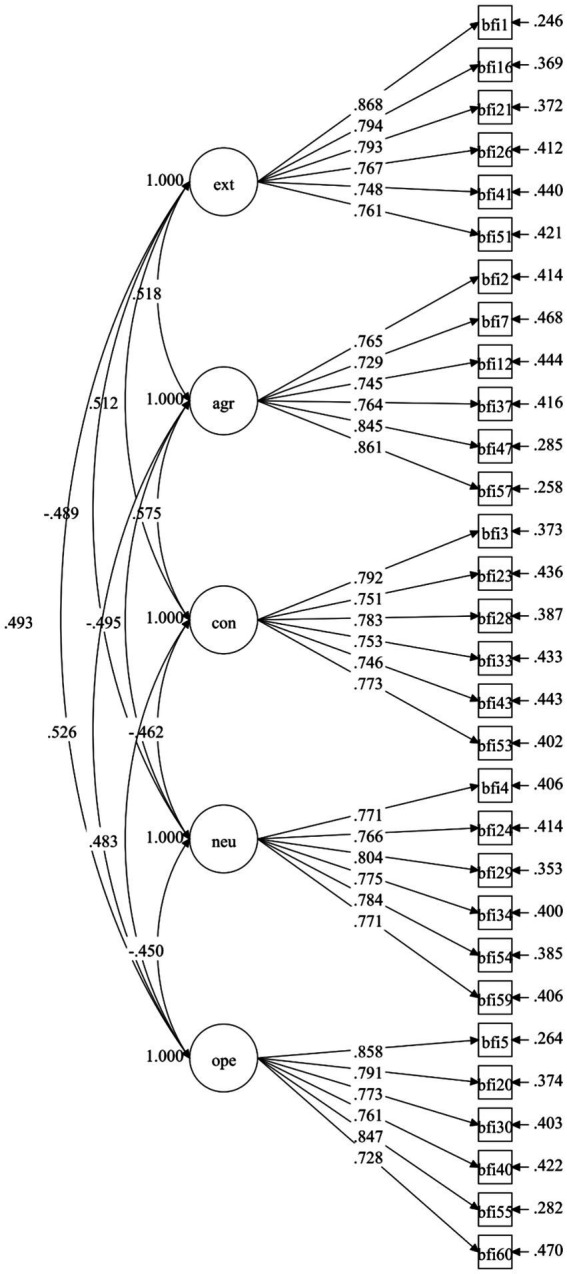
CFA diagram of the BFI-2-C short model. EXT, extraversion; AGR, agreeableness; CON, conscientiousness; NEU, negative emotionality; OPE, open-mindedness.

Both instrument versions exhibited excellent fit to the hypothesized five-factor structure (Table 3). All fit indices met or exceeded recommended thresholds, with RMSEA values below 0.06, CFI and TLI values above 0.95, and SRMR values below 0.08 (Hu and Bentler, 1999). The abbreviated version demonstrated slightly superior fit, suggesting efficient construct representation with reduced item count (Figures 1, 2).

**Table 3:** CFA fit indices for the BFI-2-C (original and short models).

Model	RMSEA (90% CI)	CFI	TLI	SRMR
Model-original	0.029 (0.026, 0.032)	0.964	0.962	0.036
Model-short	0.036 (0.031, 0.042)	0.971	0.968	0.036

RMSEA, Root Mean Square Error of Approximation; CFI, Comparative Fit Index; TLI, Tucker-Lewis Index; SRMR, Standardized Root Mean Square Residual.

### Convergent and discriminant validity

Both instrument versions demonstrated excellent reliability coefficients and validity indices. For the original format, Composite Reliability (CR) coefficients ranged from 0.923 (Agreeableness) to 0.950 (Extraversion). The abbreviated version maintained comparable CR values, ranging from 0.895 (Conscientiousness) to 0.911 (Open-Mindedness). These coefficients, all substantially exceeding the conventional threshold of 0.70, indicate robust internal consistency and suggest high coherence among items within each personality dimension (Table 4).

**Table 4:** Factor loadings of the BFI2-C for original model.

Factors/items	Factor loading	Cronbach’s alpha
Extraversion		0.948
BFI1	0.858	
BFI6	0.836	
BFI11	0.740	
BFI16	0.806	
BFI21	0.798	
BFI26	0.793	
BFI31	0.775	
BFI36	0.720	
BFI41	0.758	
BFI46	0.714	
BFI51	0.779	
BFI56	0.798	
Agreeableness		0.923
BFI2	0.767	
BFI7	0.740	
BFI12	0.744	
BFI17	0.549	
BFI22	0.629	
BFI27	0.646	
BFI32	0.594	
BFI37	0.772	
BFI42	0.656	
BFI47	0.849	
BFI52	0.636	
BFI57	0.856	
Conscientiousness		0.943
BFI3	0.808	
BFI8	0.84	
BFI13	0.748	
BFI18	0.734	
BFI23	0.762	
BFI28	0.800	
BFI33	0.770	
BFI38	0.720	
BFI43	0.700	
BFI48	0.674	
BFI53	0.782	
BFI58	0.771	
Negative emotionality		0.943
BFI4	0.778	
BFI9	0.748	
BFI14	0.750	
BFI19	0.710	
BFI24	0.765	
BFI29	0.817	
BFI34	0.794	
BFI39	0.727	
BFI44	0.730	
BFI49	0.726	
BFI54	0.793	
BFI59	0.779	
Open-mindedness		0.947
BFI5	0.854	
BFI10	0.787	
BFI15	0.817	
BFI20	0.805	
BFI25	0.709	
BFI30	0.785	
BFI35	0.717	
BFI40	0.770	
BFI45	0.735	
BFI50	0.757	
BFI55	0.849	
BFI60	0.738	

Average Variance Extracted (AVE) values for the original instrument ranged from 0.503 (Agreeableness) to 0.612 (Extraversion), while the abbreviated version demonstrated enhanced AVE coefficients ranging from 0.588 (Conscientiousness) to 0.631 (Open-Mindedness). The elevation in minimum AVE from 0.503 to 0.588 in the abbreviated version represents a particularly meaningful improvement in convergent validity parameters (Table 5).

**Table 5:** Factor loadings of the BFI2-C for short model.

Factors/items	Factor loading	Cronbach’s alpha
Extraversion		0.906
BFI1	0.868	
BFI16	0.794	
BFI21	0.793	
BFI26	0.767	
BFI41	0.748	
BFI51	0.761	
Agreeableness		0.905
BFI2	0.765	
BFI7	0.729	
BFI12	0.745	
BFI37	0.764	
BFI47	0.845	
BFI57	0.861	
Conscientiousness		0.897
BFI3	0.792	
BFI23	0.751	
BFI28	0.783	
BFI33	0.753	
BFI43	0.746	
BFI53	0.773	
Negative emotionality		0.904
BFI4	0.771	
BFI24	0.766	
BFI29	0.804	
BFI34	0.775	
BFI54	0.784	
BFI59	0.771	
Open-mindedness		0.909
BFI5	0.858	
BFI20	0.791	
BFI30	0.773	
BFI40	0.761	
BFI55	0.847	
BFI60	0.728	

Regarding discriminant validity, the highest interfactor correlation in the original instrument was observed between Agreeableness and Conscientiousness (*r* = 0.579), while in the abbreviated version, the strongest association also emerged between Agreeableness and Conscientiousness (*r* = 0.575). All interfactor correlations remained below the critical threshold of 0.85, providing robust evidence of discriminant validity. These findings confirm that each dimension within the BFI-2-C represents a distinct personality construct, an essential characteristic for multidimensional psychological assessment instruments such as the Big Five framework. Table 6 presents the comprehensive correlation matrix, CR coefficients, and AVE values for both instrument versions.

**Table 6:** Correlation matrix and discriminant validity of the BFI-2-C (original and short models).

Construct	CR.	AVE.	EXT	AGR	CON	NEU	OPE
Original							
EXT	0.950	0.612	0.782				
AGR	0.923	0.503	0.508*	0.710			
CON	0.942	0.578	0.489*	0.579*	0.760		
NEU	0.943	0.578	−0.445*	−0.461*	−0.439*	0.760	
OPE	0.948	0.606	0.438*	0.503*	0.459*	−0.388*	0.778
Short							
EXT	0.908	0.623	0.789				
AGR	0.907	0.619	0.518*	0.787			
CON	0.895	0.588	0.512*	0.575*	0.767		
NEU	0.902	0.606	−0.489*	−0.495*	−0.462*	0.779	
OPE	0.911	0.631	0.493*	0.526*	0.483*	−0.45*	0.794

*p < 0.05; CR, Composite Reliability; AVE, Average Variance Extracted; EXT, Extraversion; AGR, Agreeableness; CON, Conscientiousness; NEU, Negative Emotionality; OPE, Open-Mindedness.

### Test–retest reliability

Both versions of the BFI-2-C demonstrated strong temporal stability coefficients. The original instrument yielded Intraclass Correlation Coefficients (ICC) ranging from 0.777 (Conscientiousness) to 0.872 (Extraversion). The abbreviated version exhibited comparable or enhanced ICC values, ranging from 0.762 (Conscientiousness) to 0.942 (Negative Emotionality). Of particular note, the abbreviated version demonstrated substantial enhancement in temporal stability for Negative Emotionality (ICC increased from 0.824 to 0.942) and Open-Mindedness (ICC increased from 0.844 to 0.890). These elevated ICC values provide compelling evidence for the instrument’s reliability in capturing consistent personality attributes among Chinese adolescent football players across measurement occasions. Table 7 presents the comprehensive ICC values for both versions of the BFI-2-C.

**Table 7:** Intraclass correlation coefficients (ICC) for the BFI2-C.

Dimension	ICC original	ICC short
Extraversion	0.872	0.863
Agreeableness	0.798	0.836
Conscientiousness	0.777	0.762
Negative Emotionality	0.824	0.942
Open-Mindedness	0.844	0.890

ICC, Intraclass Correlation Coefficient.

## Discussion

This study evaluated the psychometric properties of the Chinese versions of the Big Five Inventory-2 (BFI-2-C) and its short form (BFI-2-S-C) among a cohort of adolescent football players in Shandong Province, China. The results provide robust support for the reliability, factorial validity, and temporal stability of both instrument versions, demonstrating their appropriateness for use in specialized athletic populations.

The confirmatory factor analyses (CFA) yielded excellent model fit indices for both the full and short versions of the BFI-2-C, with the abbreviated model demonstrating slightly superior parameters (e.g., CFI improvement from 0.964 to 0.971). These findings replicate and extend prior validations in general Chinese adolescent populations (Zhang et al., 2022), underscoring the structural soundness of the five-factor model even in high-performance youth sport environments.

Importantly, the strong structural integrity observed in this study supports the applicability of the Big Five framework in specialized youth sport contexts. Despite the distinctive performance demands, competitive pressures, and group-based socialization inherent in youth football, the fundamental structure of personality traits remained intact—consistent with the theoretical expectation that core personality dimensions exhibit continuity across varying social roles and environments (Allen and Laborde, 2014). However, formal invariance testing across demographic subgroups and contextual comparisons would be needed to establish measurement equivalence more rigorously.

Internal consistency was high across all five dimensions for both instruments, with Composite Reliability (CR) values far exceeding the 0.70 threshold. Notably, the abbreviated version maintained comparable reliability despite a 50% reduction in item count, supporting the contention that psychometrically sound short forms can retain measurement integrity while reducing response burden (Rammstedt and Beierlein, 2014).

Temporal stability was further established through strong test–retest reliability. The BFI-2-S-C demonstrated particularly high Intraclass Correlation Coefficients (ICCs) for Negative Emotionality and Open-Mindedness, suggesting robust stability over time even among adolescents, whose personality traits are generally in flux (Roberts and DelVecchio, 2000). These findings support the trait-like consistency of personality even during developmental periods characterized by cognitive and emotional transition.

Construct validity was confirmed through convergent and discriminant analyses. Average Variance Extracted (AVE) and CR values met or exceeded accepted benchmarks, and interfactor correlations remained below the 0.85 criterion, satisfying the Fornell-Larcker standard (Fornell and Larcker, 1981). However, convergent validity relied exclusively on internal indicators without external validation, and discriminant validity assessment did not include contemporary approaches such as HTMT (Henseler et al., 2015). Future research should incorporate external criteria and methodological advances for more comprehensive validation. The correlation patterns, especially the moderate associations between Agreeableness and Conscientiousness, are consistent with prior research in both general and sport-specific adolescent samples (Soto and John, 2017a; Allen and Laborde, 2014), potentially reflecting the socio-emotional interdependence common in collectivist youth sport settings.

The validated BFI-2-C and BFI-2-S-C offer practical and theoretical value for both researchers and practitioners. For researchers, these tools provide reliable means to investigate personality-performance relationships at both the domain and facet levels. The availability of both long and short forms facilitates methodological flexibility—enabling researchers to select instruments appropriate to their contextual constraints without compromising psychometric rigor (Soto and John, 2017a; Soto and John, 2017b).

It is important to critically evaluate this study’s contribution relative to existing BFI-2-C validation work. While Zhang et al. (2022) validated the BFI-2-C in general Chinese adolescents, our study provides incremental value in three key respects. First, we demonstrate psychometric adequacy specifically within a specialized athletic population characterized by intensive training demands and competitive pressures—contextual factors that may influence personality assessment and were not examined in prior work. Second, we provide the first validation of the 30-item short form (BFI-2-S-C) in any Chinese adolescent population, establishing its utility for time-constrained sport settings. Third, we document temporal stability through test–retest reliability, which was not assessed in the Zhang et al. validation. However, we acknowledge that our study is fundamentally a context-specific replication that confirms rather than extends the factor structure, and that more substantial incremental contributions would require examining predictive validity, measurement invariance, or facet-level functioning—areas we identify as priorities for future research.

For sport psychologists, coaches, and talent development specialists, these assessments can support individualized interventions. For instance, identifying athletes high in Conscientiousness may inform strategies to enhance training compliance, while those high in Extraversion may benefit from leadership roles. In contrast, those with elevated Negative Emotionality scores may require greater emotional regulation support (Shuai et al., 2023; MacNamara et al., 2010). These applications are especially valuable in the Chinese youth football system, where psychological tools tailored to the local sociocultural context remain limited.

### Limitations and future research

Several limitations should be acknowledged. First, the current study did not conduct measurement invariance testing across gender or age subgroups, nor did we examine the 15 facet-level traits that constitute a key advantage of the BFI-2’s hierarchical structure. The absence of invariance analyses limits our ability to justify cross-group comparisons, while the omission of facet-level assessment reduces the comprehensiveness and specificity of our validation. Future research should prioritize multi-group confirmatory factor analysis to establish measurement invariance and examine facet-level psychometric properties across gender, age, competitive level, and training intensity.

Second, our validity assessment relied exclusively on internal model-derived indicators for convergent validity and employed only the Fornell-Larcker criterion for discriminant validity. More comprehensive validation would benefit from external criteria (e.g., correlations with established personality measures, behavioral observations, sport-specific outcomes) and contemporary approaches such as HTMT (Henseler et al., 2015). Examining predictive validity for performance consistency, resilience under pressure, and team dynamics would enhance applied utility.

Third, although the sample was geographically diverse within Shandong Province, it may not generalize to athletes from other regions or sports. The study also relied exclusively on self-report methods, which may introduce social desirability biases---although the BFI-2’s balanced keying partially mitigates this risk. Future research should incorporate broader geographic and sport-type representation, peer ratings, and behavioral observations.

Finally, longitudinal designs could explore how competitive sport participation affects personality development across adolescence, and cross-cultural comparisons could illuminate how cultural factors influence personality expression in sport contexts (Cheung et al., 2011).

## Conclusion

This investigation offers strong empirical support for the use of the BFI-2-C and BFI-2-S-C among Chinese adolescent football players. Both versions demonstrated excellent structural validity, reliability, and temporal stability, confirming the cross-contextual applicability of the Big Five framework in athletic youth populations. The BFI-2-S-C, in particular, represents a psychometrically robust yet time-efficient instrument suitable for applied sport settings. These findings address a methodological gap in Chinese sports psychology and provide validated tools to support both academic inquiry and practical implementation in athlete development. Future research should expand the scope of validation to include criterion and predictive validity, examine longitudinal trait trajectories, and consider intercultural adaptations to further enrich the global applicability of personality assessment in youth sport contexts.

## Data Availability

The raw data supporting the conclusions of this article will be made available by the authors, without undue reservation.
